# Sex-Specific Association between Underlying Diseases and the Severity and Mortality Due to COVID-19 Infection: A Retrospective Observational Cohort Analysis of Clinical Epidemiological Information Collected by the Korea Disease Control and Prevention Agency

**DOI:** 10.3390/healthcare10101846

**Published:** 2022-09-23

**Authors:** Hwayeong Oh, Roeul Kim, Woojin Chung

**Affiliations:** 1 National Evidence-based Healthcare Collaborating Agency, Seoul 04933, Korea; 2 Labor Welfare Research Institute, Korea Workers’ Compensation and Welfare Service, Seoul 07254, Korea; 3 Department of Health Policy and Management, Graduate School of Public Health, Yonsei University, Seoul 03722, Korea; 4 Institute of Health Services Research, Yonsei University, Seoul 03722, Korea

**Keywords:** COVID-19, underlying disease, severity, mortality, sex, South Korea

## Abstract

This study is a retrospective observational cohort analysis aiming to explore the relationship between underlying disease and the severity and mortality rate of coronavirus disease (COVID-19) by sex. As sample subjects, 5077 confirmed COVID-19 patients were selected. The dependent variable was each patient’s clinical severity, dichotomized into two groups: clinical non-severity group and clinical severity group (including death group). Eleven underlying diseases were considered variables of interest, and each was dichotomized. Binary multivariate logistic regression model analyses were performed. Our results showed that the proportion of male patients (7.1%) in the clinical severity group was significantly higher than that of female patients (4.5%) and that the risk of being in the clinical severity group was higher in patients with specific underlying diseases. The underlying diseases varied: in males, rheumatism and autoimmune (adjusted odds ratio (aOR) = 6.69, 95% confidence interval (CI) = 1.60–27.98), dementia (aOR = 4.09, 95% CI = 2.14–7.82), cancer (aOR = 2.69, 95% CI = 1.27–5.69), and diabetes mellitus (aOR = 1.81, 95% CI = 1.18–2.77); in females, chronic kidney disease (aOR = 5.09, 95% CI = 1.87–13.86), dementia (aOR = 3.08, 95% CI = 1.18–5.23), diabetes mellitus (aOR = 1.87, 95% CI = 1.15–3.02), and hypertension (aOR = 1.73, 95% CI = 1.08–2.78). This study identified certain underlying diseases related to the high risk of being in clinically severe conditions and found that they differ between sexes. Prevention and treatment measure should be developed to reduce severity or mortality in confirmed COVID-19, based on underlying diseases and sex. However, further in-depth research is required to explore whether the findings and suggestions of this study can be generalized to other countries.

## 1. Introduction

Coronavirus disease 2019 (COVID-19) is caused by severe acute respiratory syndrome coronavirus 2 (SARS-CoV-2) [1]. The main transmission route is via respiratory droplets, which can be transmitted via coughing, sneezing, and physical contact [2]. COVID-19 has spread rapidly worldwide since its discovery in Wuhan, Hubei Province, China, in December 2019 [3]. In March 2020, the World Health Organization (WHO) declared COVID-19 a global pandemic [4]. In response to the COVID-19 pandemic, South Korea (hereafter, Korea) focused on implementing quarantine measures by raising the infectious disease crisis level from “crisis” to “serious”, mobilizing the Central Disaster and Safety Countermeasure Headquarters, and implementing social distancing [5].

Despite these measures, confirmed cases and deaths have been steadily increasing owing to the continuous spread of COVID-19 and mutant viruses. As of 12 September 2022, the cumulative number of confirmed COVID-19 cases worldwide exceeded approximately 605 million, and the death toll was approximately 6.4 million [6]. The total number of confirmed cases and deaths in Korea was approximately 24 million and 27 thousand, respectively [5].

COVID-19 poses a threat to life, health, the economy, and society [7]. Thus, it is necessary to establish protective measures, such as hand washing, wearing of masks, and isolation of high-risk groups to counteract the fatality of the disease. Moreover, closer observation and treatment of COVID-19 patients are needed to prevent these patients from developing clinically severe infections or dying [8,9]. Previous studies have reported an increased risk of clinically severe conditions and death in COVID-19 patients with specific underlying diseases. According to a study on inpatients with COVID-19 conducted in the United States, the most common underlying diseases were hypertension (46.7%), hyperlipidemia (28.9%), diabetes mellitus (27.9%), and chronic lung disease (16.1%). The underlying diseases that were significantly related to increased mortality in hospitals were metastatic solid tumors (adjusted odds ratio (aOR) = 1.57; 95% confidence interval (CI) = 1.20–2.05), myocardial infarction (aOR = 1.47; 95% CI = 1.34–1.62), and cerebrovascular disease (aOR = 1.39; 95% CI = 1.25–1.56) [10]. Another study conducted in the United Kingdom showed that underlying diseases associated with increased COVID-19 mortality were solid organ transplantation (aOR = 3.53; 95% CI = 2.77–4.49), blood cancer (aOR = 2.80; 95% CI = 2.08–3.78), and certain neurological diseases (aOR = 2.58; 95% CI = 2.38–2.79) [11]. However, these studies did not use nationally representative samples and did not include clinical symptoms as control variables. In addition, sex-specific analyses were not included.

Among Koreans, several studies have attempted to explore the association between underlying diseases and the progression of COVID-19 to death. Nevertheless, their findings seem to have limitations in explaining the severity rate. A study of 5628 confirmed COVID-19 patients in Korea showed that the risk of death is different among underlying diseases: dementia (hazard risk (HR) = 7.03), higher lymphocytes (HR = 4.66), cancer (HR = 4.27), dyspnea (HR = 3.25), and chronic obstructive pulmonary disease (HR = 3.22) [12]. However, this study conducted a univariate analysis without consideration of control variables and focused on death as a health outcome, including severe near fatal clinical conditions. Another Korean study of 8976 confirmed COVID-19 patients analyzed the difference in the proportion of those who needed specific treatment across underlying diseases such as high blood pressure, diabetes mellitus, chronic heart disease, heart failure, chronic kidney disease, and chronic obstructive pulmonary disease [13]. The study found that, compared to those without such an underlying disease, those with it were 2.7–3.3 times more likely to receive oxygen therapy and more than 4 times more likely to receive ventilator treatment. However, this study was also a univariate analysis that did not consider control variables and did not include the critical and fatal groups for whom various medical treatments were essential. Another study analyzed the difference in prognosis according to the underlying disease in 7590 patients who were confirmed to have COVID-19 until 15 May 2020 [14]. This study compared the differences between medical use (admission to intensive care units) and mortality according to 17 underlying diseases. Underlying disease was defined by selecting a diagnostic code that appeared at least once in Korea’s 2019 Health Insurance Review and Assessment Service claims data. After adjusting for age and sex, patients with diabetes mellitus and chronic complications (OR = 1.811; 95% CI = 1.241–2.642; *p* = 0.002) were associated with intensive care unit admission compared with patients without diabetes mellitus. In addition, patients with underlying congestive heart failure (OR = 1.724; 95% CI = 1.211–2.456; *p* = 0.003), dementia (OR = 1.598; 95% CI = 1.108–2.305; *p* = 0.012), diabetes mellitus with and without chronic complications (OR = 1.821; 95% CI = 1.255–2.644; *p* = 0.002 and OR = 1.518; 95% CI = 1.063–2.169; *p* = 0.022, respectively), renal disease (OR = 2.299; 95% CI = 1.371–3.858; *p* = 0.002), and malignancy (OR = 1.529; 95% CI = 1.022–2.257; *p* = 0.039) had a significantly higher risk of death than patients without them. However, the results of this study are limited mainly because this study was based on insurance claim data, not clinical data: (1) since the underlying disease was checked through claims data during 2019, it is not possible to accurately verify whether it is accompanied by the underlying disease at the time of treatment due to COVID-19; (2) there may be an error in which the diagnostic name on the insurance claim data does not accurately reflect the actual patient’s condition.

Although previous studies have linked underlying diseases to clinically severe conditions and death among confirmed COVID-19 patients, their results seem to have some limitations, as previously mentioned. Therefore, the present study aimed to explore the relationship between underlying diseases and the clinically severe condition and death among confirmed COVID-19 patients. This study used nationally representative clinical epidemiological data from the Korean Centers for Disease Control and Prevention (KCDC). In this study, the relationship between underlying diseases and a clinically severe condition and death was adjusted for demographic, health, and functional characteristics, and the relationship was compared between sexes.

## 2. Materials and Methods

### 2.1. Data Source and Study Sample

The study is a retrospective observational cohort analysis to explore the relationship between underlying disease and the severity and mortality rate of confirmed COVID-19 patients in Korea by sex. This secondary data analysis used clinical epidemiological information data, including the information of 5628 confirmed COVID-19 patients as of 30 April 2020, with each patient from hospitalization to death or release from quarantine, which was released to certified researchers by the KCDC. To prepare evidence-based measures against COVID-19, data were prepared by medical staff at COVID-19 medical sites nationwide and collected by the KCDC (Patient Information Management Group and the Korea Health Information Management Association) and the National Medical Center. Data were deliberately approved and released to the researchers after evaluating the adequacy of the disclosure of clinical epidemiological information by the COVID-19 Patient Information Disclosure Utilization Committee.

**Figure 1:**
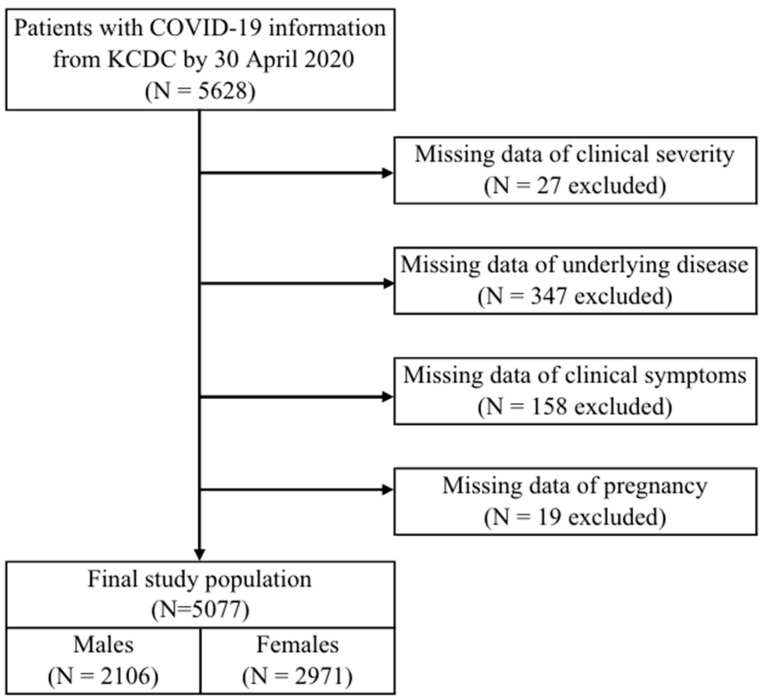
Selection process of the study sample.

Of the 5628 subjects comprising confirmed COVID-19 patients, 551 were excluded due to the lack of information about clinical severity (*n* = 27), underlying disease (*n* = 347), pregnancy (*n* = 19), and health and functional characteristics (*n* = 158) (Figure 1). In addition to maintain the representativeness of the sample, the no-answer of the body mesh index was included as one category. The final study population comprised 5077 subjects (2106 males and 2971 females).

Before conducting the analysis, we took a step to confirm whether the final study population maintained the characteristics after exclusion and was deemed representative of the population. For this purpose, a homogeneity test was performed on sex and age variables using the chi-squared test. A *p*-value of <0.05 was regarded as statistically significant. The results confirmed that sex and age were homogeneous before and after the exclusion. The Research Deliberation Committee of Yonsei Medical Center approved this study for exemption from deliberation and consent (task number: 4–2021–0676).

### 2.2. Measurements

#### 2.2.1. Dependent Variable: Clinical Severity

The KCDC classified confirmed COVID-19 patients into four categories based on their symptoms and health outcomes: (1) mild or less, (2) moderate, (3) severe, and (4) dead. The ‘mild or less’ category included patients with no activity limitation or those with limitations in activity but not requiring oxygen treatment. ‘Moderate’ category included symptoms requiring oxygen treatment with nasal prong or facial mask. ‘Severe’ category included patients with chronic renal failure or those requiring non-invasive ventilation, high-flow oxygen therapy, invasive ventilation, or multi-organ oxygen supply. According to the KCDC’s COVID-19 Response Guidelines (No. 7), patients classified as ‘severe’ were assigned to state-designated inpatient treatment beds such as tertiary care hospitals or general hospitals that could treat critically ill patients.

**Figure 2:**
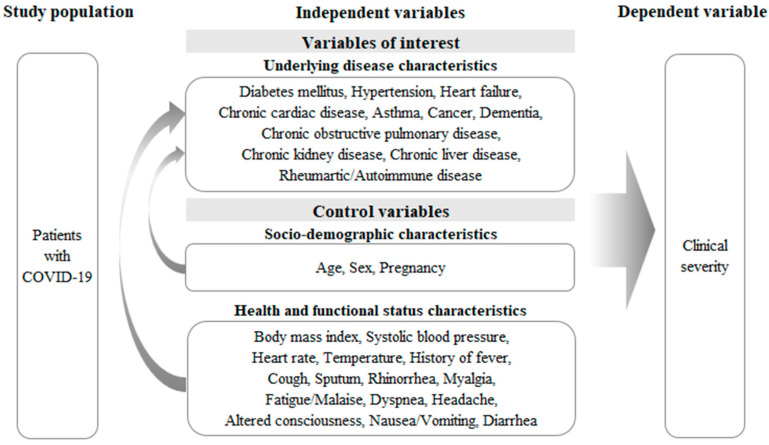
Study framework.

In the present study, we divided the confirmed COVID-19 patients into two groups: “the clinical severity group” when they were classified as either “severe” or “dead”; and “the clinical non-severity group” when they were classified as either “mild or less” or “moderate”. For each patient, a dichotomous dependent variable was constructed with 1 when the patient belonged to the clinical severity group and 0 when the patient belonged to the clinical non-severity group (Figure 2).

#### 2.2.2. Variables of Interest: Underlying Diseases

Underlying diseases (or comorbidities) were chronic diseases that were present in confirmed COVID-19 patients. The KCDC provided information on underlying diseases in the form of binary variables for each of the 11 underlying diseases: diabetes mellitus, high blood pressure, heart failure, chronic heart disease, asthma, chronic obstructive pulmonary disease, chronic kidney disease, chronic liver disease, cancer, rheumatism/autoimmune disease, and dementia (Figure 2).

#### 2.2.3. Control Variables

This study used demographic, health, and functional characteristics as control variables. The demographic characteristics included sex, age, and pregnancy status. Health and functional characteristics included initial examination findings, body mass index, systolic blood pressure, pulse, body temperature, and presence of symptoms such as sore throat, runny nose, fatigue/wornness, headache, and nausea/diarrhea (Figure 2).

### 2.3. Statistical Analysis

Our analysis consisted of three parts. The first part involved descriptive analyses to understand the characteristics of the study subjects, which were expressed as frequencies and percentages. The second part involved univariate analyses to examine characteristics related to clinical severity, where the chi-square test, Fisher’s exact test, and Firth’s correction were conducted. Finally, in the third part, we performed multivariate binary logistic regression analyses to explore the underlying diseases related to clinical severity using crude models with no control variables and adjusted models with all-studied control variables.

Before embarking on a detailed analysis, we considered three issues: stratification by sex, variable selection in multivariate analyses, and reduction/elimination of multicollinearity in multivariate analyses.

First, regarding stratification by sex, we found that previous studies confirmed a difference in mortality due to COVID-19 between sexes [11,15]. In addition, as a pilot analysis, the present study revealed that 7.5% of male and 4.6% of female patients were classified into the clinical severity group. Therefore, since these studies implicated a sex difference in the clinical severity of COVID-19, we decided to conduct all analyses by classifying the sample population by sex and comparing the accompanying results between sexes.

Second, regarding variable selection in multivariate analyses, we excluded some independent variables with no observations found in the clinical severity group of the dependent variable. We then applied the stepwise variable selection method, where the significance level was SLE = 0.20 and SLS = 0.20, to variables pertaining to demographic, health, and functional characteristics.

Third, for the independence test between all the selected variables in the multivariate analyses, multicollinearity was checked using the following three-step procedure:

We obtained variance inflation factors among all variables and found that they were between 1.02 and 1.56 in males and between 1.01 and 1.52 in females, respectively. In the second step, since all variables in this study were categorical, we performed cross-table analyses among each pair of all control variables and variables of interest. The results were compared and analyzed using the chi-square test and Fisher’s exact test. In the third step, the correlation intensity between variables with a significant difference in the cross-table analysis was compared and analyzed. In this case, the phi correlation coefficient was checked between variables with two categories, and the correlation size was checked between variables with three or more categories, using Cramer’s V coefficient. Finally, after undergoing the process of variable selection and the reduction/elimination of multicollinearity, we conducted a multivariate analysis with the final selected variables, estimating the odds ratios (ORs) and 95% CIs.

A *p*-value < 0.05 was used to test the statistical validity of each model and confirm its suitability. SAS software (version 9.4; SAS Institute, Cary, NC, USA) was used for all the statistical analyses.

## 3. Results

Among the 5077 study subjects, 4794 patients (94.4%) were categorized into the clinical non-severity group and 283 patients (5.6%) into the clinical severity group, with 7.1% male patients and 4.5% female patients. Table 1 shows the distributions of demographic characteristics, health and functional characteristics, and underlying diseases as frequencies and percentages for the study participants by sex.

Table 2 displays the findings from the univariate analyses examining the difference in clinical severity in terms of frequency and proportion according to demographic characteristics, health and functional characteristics, and underlying diseases.

In particular, the underlying diseases that showed significant differences in clinical severity differed by sex. Male patients had diabetes mellitus, high blood pressure, heart failure, chronic heart disease, chronic obstructive pulmonary disease, chronic kidney disease, cancer, rheumatism, autoimmune disease, and dementia. However, female patients had diabetes mellitus, high blood pressure, heart failure, chronic heart disease, asthma, chronic obstructive pulmonary disease (COPD), chronic kidney disease, and dementia.

Table 3 shows the results of the multivariate logistic regression analyses examining the crude and adjusted associations between underlying diseases and clinical severity by sex.

In male patients, the C-statistic values in the crude and adjusted models were 79.4% and 90.3%, respectively, and the AIC (Akaike’s Information Criterion) statistic values in both models were 917.3 and 699.2 in the crude and adjusted models, respectively. This indicates that the adjusted model is more suitable than the crude model for explaining the relationship between the underlying diseases and clinical severity. Although the four underlying diseases (hypertension, heart failure, chronic cardiac disease, and chronic obstructive pulmonary disease) were significant in the crude model, they were not significant in the adjusted model. However, diabetes mellitus, cancer, rheumatic/autoimmune diseases, and dementia were significant in both the crude and adjusted models. In the adjusted model, compared to patients with no underlying disease, the risk of belonging to the clinical severity group was higher than those with the following diseases: diabetes mellitus (aOR = 1.81, 95% CI = 1.18–2.77), cancer (aOR = 2.69, 95% CI = 1.27–5.69), rheumatism and autoimmune disease (aOR = 6.69, 95% CI = 1.60–27.98), and dementia (aOR = 4.09, 95% CI = 2.14–7.82).

Similar to male patients, in female patients, the adjusted model seemed to be more suitable than the crude model for explaining the relationship between underlying diseases and clinical severity. The c-statistic value of the adjusted model, 92.6%, was larger than that of the crude model (82.5%), and the AIC statistic of the adjusted model (691.8) was lower than that of the crude model (869.1). Among female patients, heart failure and asthma, which were significant in the crude model, were not significant in the adjusted model. However, whether the model was adjusted for diabetes mellitus, hypertension, chronic kidney disease, and dementia was significantly associated with the clinical severity. In the adjusted model, relative to patients with no underlying disease, the risk of belonging to the clinical severity group was higher than those with the following underlying diseases: diabetes mellitus (aOR = 1.87, 95% CI = 1.15–3.02), hypertension (aOR = 1.73, 95% CI = 1.08–2.78), chronic kidney disease (aOR = 5.09, 95% CI = 1.87–13.86), and dementia (aOR = 3.08, 95% CI = 1.18–5.23).

Regarding control variables, in male patients, a high risk of belonging to the clinical severity group was found in the following groups: older patients, patients with tachycardia, patients whose body mass information was not reported, patients with fever, patients with sputum, patients with no rhinorrhea, or patients with altered consciousness. For female patients, a high risk of belonging to the clinical severity group was shown in the following groups: patients belonging to the highest age group (≥70 years), patients with fever, patients having no sore throat, patients with dyspnea, patients having no headache, or patients with altered consciousness.

## 4. Discussion

The present study explored the association between underlying diseases and the clinical severity of confirmed COVID-19 patients by sex, adjusted for demographic, health, and functional characteristics.

This study revealed that although there were fewer males (41.5%) than females (58.5%) among the confirmed COVID-19 patients, male patients had a higher proportion in the clinical severity group (7.1%) than female patients (4.5%). This is consistent with the result of a previous study in which the ratio of the severe group due to COVID-19 to its non-severe group was higher in male patients than in female patients [15].

According to the results of the adjusted models, for the underlying diseases associated with a higher risk of belonging to the clinical severity group due to COVID-19 compared to those with no underlying diseases, some underlying diseases were the same in both male and female patients, but others were different between the sexes. Males were diagnosed with diabetes mellitus, cancer, rheumatism/autoimmune diseases, and dementia, and females were diagnosed with diabetes mellitus, high blood pressure, chronic kidney disease, and dementia.

Irrespective of sex, diabetes mellitus and dementia were associated with clinical severity due to COVID-19. This finding is in line with the results of previous studies that diabetes mellitus is associated with severity, complications, or mortality in confirmed COVID-19 patients [16,17] and that dementia is a risk factor for death in COVID-19 patients [18].

For male patients, the risk of belonging to the clinical severity group of COVID-19 was higher in patients with cancer and rheumatism/autoimmune diseases than in those with no underlying disease characteristics. This is supported by previous studies on cancer [19], and rheumatoid or autoimmune diseases [15,20]. In addition, chemotherapy, radiation therapy, and immunotherapy performed within the last two weeks have been reported to be very important in predicting the severity or fatality of confirmed COVID-19 patients [21]. Unfortunately, to date, no study has reported why cancer and rheumatism/autoimmune diseases are risk factors for being “severe or dead” in males but not in females. Therefore, additional detailed studies focusing on these sex-based differences are required.

For female patients, hypertension was a risk factor for clinical severity due to COVID-19. This is consistent with previous studies showing that hypertension is associated with the severity of COVID-19 and subsequent death [15,22]. However, no previous study has reported a lack of statistical significance in males. Therefore, these sex differences might be due to a difference in sex or a sex difference in the use of pharmaceuticals for hypertension. Further studies, including a closer examination of this topic, are necessary. 

In addition, in female patients, chronic kidney disease was a risk factor for severe or dead” due to COVID-19. This finding is similar to that of previous studies showing that chronic kidney disease is associated with the severity and mortality of COVID-19 [23]. Unlike other patients, patients with chronic kidney disease, especially those undergoing dialysis, are vulnerable to viral and bacterial infections and are thus more likely to be exposed to the hospital environment regardless of their severity. Therefore, the risk of infection in these patients cannot be ruled out. However, in this study, we could not include whether the patients under investigation received dialysis and if any, which were the types of dialysis they received; instead, we only investigated whether the chronic renal disease was diagnosed. This lack of sufficient information, such as the practice and type of dialysis, might cause sex differences.

As shown in the results of this study, it is similar to the results of a U.S. study that shows a high risk of severity and complications such as ‘death’, ‘invasive ventilator treatment’, and ‘admission to intensive care’ compared to none of the COVID-19 patients with underlying diseases [24]. An analysis of the underlying diseases of deaths from recent Korean statistical data confirmed the difference between patients with underlying diseases (98.9%) and patients without underlying diseases (1.1%) among 901 people who completed the underlying disease survey among total deaths: 1183 people. The underlying diseases were high blood pressure 39.3%, dementia 30.7%, and diabetes 23.1% [25]. As such, as a study on underlying diseases that affect clinical severity, the results of this study confirmed through sex comparison analysis of underlying diseases and clinical severity in Korea can be the basis for specific sex-based diseases.

When discussing the associations between control and dependent variables, it is important to note that all control variables were based on clinical opinions at the time of hospitalization. Therefore, if a patient was hospitalized when they were already in a clinically severe condition, the control variables regarding health and functional characteristics could be the outcome of the condition.

Regarding age, male and female patients aged >70 years were more likely to belong to the clinically severe group than to the other age groups. This is similar to a previous study in which older people were at high risk of having severe and fatal COVID-19 [15]. In addition, male patients were more likely to belong to the clinical severity group when their pulse was in the tachycardia group. This is supported by the result of a previous study showing that tachycardia is a characteristic associated with the prognosis of severe COVID-19 [26].

In this study, the distribution of clinical severity was not high in children and young adults (0–39 years). However, previous U.S. studies showed that children under the age of 18 with underlying diseases had a higher risk of hospitalization and a higher risk of developing severe infections in hospitalized patients than children without underlying diseases [27]. In addition, in recent Korean statistical data, 44 deaths of children and adolescents under the age of 18 were cumulative on 18 August 2022, and underlying diseases were confirmed in 52.3% of the deaths [25]. Therefore, there is a need for this study for the population aged 0–39 including children and adolescents, so all ages including the population of children and young adults (0–39 years) were targeted. However, this study has a low distribution of clinical severity as data on the initial period of COVID-19, so further studies are needed in the future using population data including many children and adolescents for this study.

Both male and female patients had a high risk of belonging to the clinically severe group when they had a fever or altered consciousness. This is similar to previous studies showing that the association between fever and death in patients with COVID-19 increases significantly with body temperature [28] and that patients with altered consciousness are at a higher risk of death than that of those who do not [29]. Furthermore, among them, both male and female patients showed considerable differences in the risk of belonging to the clinical severity group between those with and without altered consciousness (males aOR = 86.15, 95% CI = 7.66–969.16, females aOR = 20.03, 95% CI = 5.40–74.30). Hence, whether a COVID-19 patient has altered consciousness at the time of hospitalization may be an important predictor of whether the patient will proceed to the “severe or dead” condition.

In addition, a high risk of belonging to the clinically severe group was found in male patients with sputum or those without rhinorrhea, and an increased risk was shown in female patients without a sore throat. Sputum, rhinorrhea, and sore throat are known symptoms of respiratory infections. Without further detailed studies confirming the association among these symptoms, severe COVID-19 infection and their differences between sexes would be difficult. The present study showed that in female patients, those with headaches were associated with a lower risk of being in the “severe or dead” condition than that of those without. Although previous studies showed that headaches could appear as symptoms of COVID-19 infection or neurological conditions or complications [30,31], it was difficult to determine the underlying reason for this occurrence in prior studies of COVID-19 patients. Among female patients, those with dyspnea showed a higher risk of belonging to the clinical severity group than those without. This is supported by a previous study showing that dyspnea is more common in patients who die than in those who recover [32].

The results of this study have several policy implications. First, the underlying diseases associated with a high risk of belonging to the clinical severity group due to COVID-19 infection were diabetes mellitus and dementia in both male and female patients; cancer, rheumatism, and autoimmune diseases in male patients; and high blood pressure and chronic kidney diseases in female patients. Therefore, it seems necessary to develop and provide customized programs for preventing COVID-19 infection and its progression to severe or fatal conditions according to people’s underlying diseases and sex.

Second, aside from the underlying diseases, a high risk of belonging to the clinically severe group due to COVID-19 infection was found in people such as those aged ≥70 years, those with fever, or those with altered consciousness at the time of hospitalization. At the time of hospitalization, these patients were classified into the high-risk group. Therefore, if necessary, it is required to introduce a patient management system for these high-risk groups, such as careful observation of the patient’s condition and appropriate treatment. Through this, the social and economic costs for such patients may be reduced, and their loss of health and life may be reduced.

Third, the prospects for economic and social losses due to the COVID-19 mutant virus and breakthrough infection are dim [33,34]. In addition to the present study, policy support for further research is needed to identify COVID-19 patients with a high risk of belonging to the clinical severity group at the earliest to prepare measures for the prevention and treatment of COVID-19 and manage medical resources efficiently.

### 4.1. Strengths

This study has several strengths. First, to the best of our knowledge, this study is the first to examine the sex-specific associations between the underlying diseases and the risk of being in clinically severe conditions among confirmed COVID-19 patients, using nationally representative clinical epidemiological information data provided by the KCDC, and conducting multivariate regression model analyses adjusted for demographic, health, and functional characteristics after considering variable selection and multicollinearity reduction. Second, this study identified certain underlying diseases related to the high risk of being in clinically severe conditions and found that they differ between sexes.

### 4.2. Limitations

This study had some limitations. First, because the data of this study were cross-sectional, no causal relationship between the dependent and independent variables could be established. In particular, regarding a reverse causality problem, some control variables such as fever might be subject to such a problem because control variables were reported based on the time of hospitalization; however, variables of interest, underlying diseases, are not likely to be affected by such a problem because underlying diseases occurred before COVID-19 infection. Further prospective studies are needed for a more detailed analysis. Second, although this study used the most recent data provided by the KCDC at the time of study planning, the participants were confirmed to have died by 30 April 2020. Further studies are needed, including patients infected by recent COVID-19-mutated viruses. In addition, until 30 April 2020, when the data of this study were collected, vaccination had not yet been implemented. Accordingly, it is difficult to fully extrapolate the results of this study to the current situation where vaccination is being performed. Therefore, in the future, it is necessary to conduct additional research by applying recent data after vaccination of COVID-19 was implemented in Korea. Third, the data provided by the KCDC included only the presence or absence of a diagnosis of underlying diseases for each participant. Information on the severity, drug administration, and administration history for each underlying disease is lacking. Some studies reported the relationship between the risks of being in the “severe or dead” group among confirmed COVID-19 patients and the severity status of other underlying diseases such as heart failure, cardiomyopathy, cardiac arrhythmia, and severity of COVID-19 [21,35,36]. Further research is needed to obtain detailed information regarding each underlying disease. Fourth, the data of this study were de-identified, and most of the variables were provided as categorical data. For example, the age group variables were classified as categorical data. This leads to limitations in analyzing each life cycle of the population. If raw data are provided in the future, this will enable a more detailed analysis. Fifth, this study classified people without data on BMI into one category and included them in the analysis. The reason is that if we excluded 1084 people (about 21%) who did not have BMI data among 5077 subjects, it would be difficult to maintain the representativeness of the study sample. As well-known, in difficult situations such as unconsciousness in the medical field, physical measurement is difficult, and eventually, people with high clinical severity are at risk of being excluded from the data. According to our additional analysis, the rate of clinical severity of people without BMI data was the second highest for both men and women after the underweight group. Finally, although this study addressed the sex differences in the association between underlying diseases and clinical severity among confirmed COVID-19 patients, it was very unfortunate to not explore the reason underlying the sex difference. This may be attributed to the lack of information in the data we could find and use. For example, we could not include health behavior characteristics such as smoking status as control variables. Some studies have shown that severity and mortality due to COVID-19 are associated with smoking status [37,38]. Considering that in Korea, smoking prevalence is much higher in males than in females, the absence of a smoking variable might result in confounding the sex difference in the association between the underlying diseases and the risk of belonging to the clinical severity group among COVID-19 patients.

## 5. Conclusions

Presently, considering that there has been no study on the association between underlying diseases and clinical severity due to COVID-19, this is the first study to investigate the association by sex using a multivariate regression analysis of a nationally representative dataset in Korea. The present study’s findings suggest that prevention and treatment measures to reduce the risk of clinically severe conditions among confirmed COVID-19 patients should be established considering patients’ underlying diseases and sex. The way is necessary to provide preventive education by classifying the underlying disease factors related to the clinical severity of COVID-19 infection into a high-risk group and to provide appropriate treatment through careful observation during the initial treatment. The results of this study will serve as basic data for health and medical policy, which can contribute to protecting the health and life of the people and reducing economic and social losses through efficient management of medical resources in Korea. However, further in-depth research is required to explore whether the findings and suggestions of this study can be generalized to other countries.

## Figures and Tables

**Table 1:** General characteristics of the study sample by sex.

Variable	Category	Males (*N* = 2106)		Females (*N* = 2971)	
		*N*	%	*N*	%
Socio-demographic characteristics					
Age (years)	0–39	853	(40.5)	848	(3.5)
	40–69	929	(44.1)	1604	(54.0)
	≥70	324	(15.4)	519	(17.5)
Pregnancy	No	-	-	2952	(99.4)
	Yes	-	-	19	(0.6)
Health and functional status characteristics					
Body mass index	Underweight	73	(3.5)	155	(5.2)
	Normal	564	(26.8)	1111	(37.4)
	Overweight	452	(21.4)	493	(16.6)
	Obese	610	(29.0)	535	(18.0)
	No-answer	407	(19.3)	677	(22.8)
Systolic blood pressure	Normal	361	(17.2)	874	(29.4)
	Pre-hypertension	902	(42.8)	1162	(39.1)
	Hypertension	843	(40.0)	935	(31.5)
Heart rate	Bradycardia	49	(2.3)	59	(2.0)
	Normal	1748	(83.0)	2510	(84.5)
	Tachycardia	309	(14.7)	402	(13.5)
Fever	No	1621	(77.0)	2248	(75.7)
	Yes	485	(23.0)	723	(24.3)
Cough	No	1275	(60.5)	1671	(56.2)
	Yes	831	(39.5)	1300	(43.8)
Sputum	No	1565	(74.3)	2053	(69.1)
	Yes	541	(25.7)	918	(30.9)
Sore throat	No	1849	(87.8)	2451	(82.5)
	Yes	257	(12.2)	520	(17.5)
Rhinorrhea	No	1905	(90.5)	2666	(89.7)
	Yes	201	(9.5)	305	(10.3)
Myalgia	No	1817	(86.3)	2438	(82.1)
	Yes	289	(13.7)	533	(17.9)
Fatigue Malaise	No	2007	(95.3)	2843	(95.7)
	Yes	99	(4.7)	128	(4.3)
Dyspnea	No	1866	(88.6)	2596	(87.4)
	Yes	240	(11.4)	375	(12.6)
Headache	No	1835	(87.1)	2397	(80.7)
	Yes	271	(12.9)	574	(19.3)
Altered consciousness	No	2094	(99.4)	2954	(99.4)
	Yes	12	(0.6)	17	(0.6)
Nausea Vomiting	No	2040	(96.9)	2802	(94.3)
	Yes	66	(3.1)	169	(5.7)
Diarrhea	No	1931	(91.7)	2707	(91.1)
	Yes	175	(8.3)	264	(8.9)
Underlying diseases characteristics					
Diabetes mellitus	No	1794	(85.2)	2622	(88.2)
	Yes	312	(14.8)	349	(11.8)
Hypertension	No	1633	(77.5)	2308	(77.7)
	Yes	473	(22.5)	663	(22.3)
Heart failure	No	2086	(99.1)	2935	(98.8)
	Yes	20	(0.9)	36	(1.2)
Chronic cardiac disease	No	2020	(95.9)	2885	(97.1)
	Yes	86	(4.1)	86	(2.9)
Asthma	No	2060	(97.8)	2896	(97.5)
	Yes	46	(2.2)	75	(2.5)
Chronic obstructive pulmonary disease	No	2082	(98.9)	2956	(99.5)
	Yes	24	(1.1)	15	(0.5)
Chronic kidney disease	No	2080	(98.8)	2943	(99.1)
	Yes	26	(1.2)	28	(0.9)
Chronic liver disease	No	2060	(97.8)	2937	(98.9)
	Yes	46	(2.2)	34	(1.1)
Cancer	No	2057	(97.7)	2880	(96.9)
	Yes	49	(2.3)	91	(3.1)
Rheumatic Autoimmune disease	No	2094	(99.4)	2946	(99.2)
	Yes	12	(0.6)	25	(0.8)
Dementia	No	2038	(96.8)	2820	(94.9)
	Yes	68	(3.2)	151	(5.1)

**Table 2:** Differences in the clinical severity for each sample characteristic by sex.

Variable	Category	Males				*p*-Value	Females				*p*-Value
		Clinical Non-Severity (*N* = 1956)		Clinical Severity (*N* = 150)			Clinical Non-Severity (*N* = 2838)		Clinical Severity (*N* = 133)		
Socio-demographic characteristics											
Age (years)	0–39	850	(99.6)	3	(0.4)	<0.001	844	(99.5)	4	(0.5)	<0.001
	40–69	874	(94.1)	55	(5.9)		1578	(98.4)	26	(1.6)	
	≥70	232	(71.6)	92	(28.4)		416	(80.2)	103	(19.8)	
Pregnancy	No						2819	(95.5)	133	(4.5)	0.677
	Yes						19	(100.0)	-	-	
Health and functional status characteristics											
Body mass index	Underweight	67	(91.8)	6	(8.2)	<0.001	143	(92.3)	12	(7.7)	<0.001
	Normal	533	(94.5)	31	(5.5)		1078	(97.0)	33	(3.0)	
	Overweight	430	(95.1)	22	(4.9)		483	(98.0)	10	(2.0)	
	Obese	568	(93.1)	42	(6.9)		514	(96.1)	21	(3.9)	
	No-answer	358	(88.0)	49	(12.0)		620	(91.6)	57	(8.4)	
Systolic blood pressure	Normal	332	(92.0)	29	(8.0)	0.032	832	(95.2)	42	(4.8)	<0.001
	Pre-hypertension	853	(94.6)	49	(5.4)		1130	(97.2)	32	(2.8)	
	Hypertension	771	(91.5)	72	(8.5)		876	(93.7)	59	(6.3)	
Heart rate	Bradycardia	48	(98.0)	1	(2.0)	<0.001	54	(91.5)	5	(8.5)	0.173
	Normal	1640	(93.8)	108	(6.2)		2404	(95.8)	106	(4.2)	
	Tachycardia	268	(86.7)	41	(13.3)		380	(94.5)	22	(5.5)	
Fever	No	1538	(94.9)	83	(5.1)	<0.001	2163	(96.2)	85	(3.8)	0.001
	Yes	418	(86.2)	67	(13.8)		675	(93.4)	48	(6.6)	
Cough	No	1184	(92.9)	96	(7.1)	0.974	1589	(95.1)	82	(4.9)	0.198
	Yes	772	(92.9)	59	(7.1)		1249	(96.1)	51	(3.9)	0.198
Sputum	No	1469	(93.9)	96	(6.1)	0.003	1958	(95.4)	95	(4.6)	0.552
	Yes	487	(90.0)	54	(10.0)		880	(95.9)	38	(4.1)	
Sore throat	No	1709	(92.4)	140	(7.6)	0.032	2321	(94.7)	130	(5.3)	<0.001
	Yes	247	(96.1)	10	(3.9)		517	(99.4)	3	(0.6)	
Rhinorrhea	No	1759	(92.3)	130	(7.2)	0.886	2537	(95.2)	129	(4.8)	0.005
	Yes	197	(98.0)	20	(6.9)		301	(98.7)	4	(1.3)	
Myalgia	No	1687	(92.8)	130	(7.2)	0.002	2315	(95.0)	123	(5.0)	0.001
	Yes	269	(93.1)	20	(6.9)		523	(98.1)	10	(1.9)	
Fatigue Malaise	No	1872	(93.3)	135	(6.7)	0.002	2717	(95.6)	126	(4.4)	0.579
	Yes	84	(84.8)	15	(15.2)		121	(94.5)	7	(5.5)	
Dyspnea	No	1794	(96.1)	72	(3.9)	<0.001	2524	(97.2)	72	(2.8)	<0.001
	Yes	162	(67.5)	78	(32.5)		314	(83.7)	61	(16.3)	
Headache	No	1699	(92.6)	136	(7.4)	0.180	2272	(94.8)	125	(5.2)	<0.001
	Yes	257	(94.8)	14	(5.2)		566	(98.6)	8	(1.4)	
Altered consciousness	No	1955	(93.4)	139	(6.6)	<0.001	2831	(95.8)	123	(4.2)	<0.001
	Yes	1	(83.3)	11	(91.7)		7	(41.2)	10	(58.8)	
Nausea Vomiting	No	1901	(93.2)	139	(6.8)	0.006	2677	(95.5)	125	(4.5)	0.868
	Yes	55	(83.3)	11	(16.7)		161	(95.3)	8	(4.7)	
Diarrhea	No	1792	(92.8)	139	(7.2)	0.653	2585	(95.5)	122	(4.5)	0.799
	Yes	164	(93.7)	11	(6.3)		253	(95.8)	11	(4.2)	
Underlying diseases characteristics											
Diabetes mellitus	No	1702	(94.9)	92	(5.1)	<0.001	2536	(96.7)	86	(3.3)	<0.001
	Yes	254	(81.4)	58	(18.6)		302	(86.5)	47	(13.5)	
Hypertension	No	1560	(95.5)	73	(4.5)	<0.001	2263	(98.1)	45	(1.9)	<0.001
	Yes	396	(83.7)	77	(16.3)		575	(86.7)	88	(13.3)	
Heart failure	No	1942	(93.1)	144	(6.9)	0.002	2814	(95.9)	121	(4.1)	<0.001
	Yes	14	(70.0)	6	(30.0)		24	(66.7)	12	(33.3)	
Chronic cardiac disease	No	1888	(93.5)	132	(6.5)	<0.001	2762	(95.7)	123	(4.3)	0.004
	Yes	68	(79.1)	18	(20.9)		76	(88.4)	10	(11.6)	
Asthma	No	1914	(92.9)	146	(7.1)	0.565	2772	(95.7)	124	(4.3)	0.006
	Yes	42	(91.3)	4	(8.7)		66	(88.0)	9	(12.0)	
Chronic obstructive pulmonary disease	No	1938	(93.1)	144	(6.9)	0.005	2.827	(95.9)	122	(4.1)	0.004
	Yes	18	(75.0)	6	(25.0)		17	(60.7)	11	(39.3)	
Chronic kidney disease	No	1937	(93.1)	143	(6.9)	0.002	2805	(95.5)	132	(4.5)	<0.001
	Yes	19	(73.1)	7	(26.9)		33	(97.1)	1	(2.9)	
Chronic liver disease	No	1916	(93.0)	144	(7.0)	0.137	2751	(95.5)	129	(4.5)	0.498
	Yes	40	(8.7)	6	(13.0)		87	(95.6)	4	(4.4)	
Cancer	No	1921	(93.4)	136	(6.6)	<0.001	2751	(95.5)	129	(4.5)	0.858
	Yes	35	(71.4)	14	(28.6)		87	(95.6)	4	(4.4)	
Rheumatic Autoimmune disease	No	1947	(93.0)	147	(7.0)	0.048	2813	(95.5)	133	(4.5)	0.626
	Yes	9	(75.0)	3	(25.0)		25	(100.0)	-	-	
Dementia	No	1917	(94.1)	121	(5.9)	<0.001	2734	(97.0)	86	(3.0)	<0.001
	Yes	39	(57.4)	29	(42.6)		104	(68.9)	47	(31.1)	

**Table 3:** The associations between underlying diseases and the clinical severity by sex.

Variables	Males				Females			
	Crude ^a^		Adjusted ^b^		Crude ^a^		Adjusted ^b^	
	OR	95% CI	OR	95% CI	OR	95% CI	OR	95% CI
Underlying diseases (ref: No disease)								
Diabetes mellitus	2.59 ***	(1.72–3.89)	1.81 **	(1.18–2.77)	1.93 **	(1.24–2.99)	1.87 *	(1.15–3.02)
Hypertension	2.59 ***	(1.76–3.81)	1.20	(0.79–1.82)	4.06 ***	(2.66–6.20)	1.73 *	(1.08–2.78)
Heart failure	3.57 *	(1.20–10.61)	1.78	(0.55–5.74)	3.19 **	(1.34–7.59)	2.04	(0.82–5.04)
Chronic cardiac disease	2.11 *	(1.14–3.92)	1.36	(0.68–2.72)	1.09	(0.48–2.45)	1.01	(0.42–2.46)
Asthma	0.94	(0.30–2.92)	0.68	(0.18–2.64)	2.88 *	(1.22–6.76)	1.78	(0.70–4.48)
Chronic obstructive pulmonary disease	3.31 *	(1.08–10.09)	2.55	(0.84–7.78)	1.51	(0.34–6.74)	1.27	(0.29–5.59)
Chronic kidney disease	1.73	(0.60–4.98)	1.77	(0.60–5.26)	5.62 ***	(2.25–14.06)	5.09 ***	(1.87–13.86)
Chronic liver disease	1.09	(0.41–2.88)	1.22	(0.45–3.32)	0.54	(0.07–4.19)	0.56	(0.07–4.46)
Cancer	5.47 ***	(2.71–11.04)	2.69 **	(1.27–5.69)	0.89	(0.29–2.70)	0.86	(0.25–2.90)
Rheumatic/Autoimmune disease	4.81 *	(1.16–19.98)	6.69 **	(1.60–27.98)	-	-	-	-
Dementia	10.44 ***	(5.99–18.20)	4.09 ***	(2.14–7.82)	7.33 ***	(4.67–11.51)	3.08 ***	(1.81–5.23)
Age (ref: 0–39)								
40–69			8.80 ***	(13.81–127.38)			2.21	(0.74–6.56)
≥70			41.94 ***				11.84 ***	(3.97–35.31)
Body mass index (ref: Normal)								
Underweight			1.32	(0.43–4.07)			-	-
Overweight			1.08	(0.57–2.04)			-	-
Obese			1.73	(0.98–3.07)			-	-
No-answer			1.89*	(1.07–3.34)			-	-
Heart rate (ref: Normal)								
Bradycardia			0.55	(0.10–3.11)			-	-
Tachycardia			2.21 **	(1.37–3.57)			-	-
Fever (Ref: No)			2.53 ***	(1.68–3.82)			2.27 ***	(1.43–3.60)
Cough (Ref: No)			0.88	(0.54–1.42)			-	-
Sputum (Ref: No)			1.74 *	(1.06–2.86)			-	-
According to our			-	-			0.17 **	(0.05–0.59)
Rhinorrhea (Ref: No)			0.35 *	(0.13–0.93)			-	-
Myalgia (Ref: No)			-	-			0.51	(0.24–1.07)
Dyspnea (Ref: No)			-	-			6.43 ***	(4.07–10.15)
Headache (Ref: No)			0.90	(0.47–1.72)			0.36 *	(0.16–0.83)
Altered consciousness (Ref: No)			86.15 ***	(7.66–969.16)			20.03 ***	(5.40–74.30)
Nausea/Vomiting (Ref: No)			-	-			0.70	(0.28–1.76)

Note: OR, odds ratio; CI, confidence interval; ***, **, and * represent 0.001, 0.01, and 0.05, respectively. **^a^** Model with no covariate. **^b^** Model with such covariates as age, body mass index, heart rate, fever, cough, sputum, rhinorrhea, headache, and altered consciousness for males and age, fever, sore throat, myalgia, dyspnea, headache, altered consciousness, and nausea/vomiting for females.

## Data Availability

Data from authors will be available upon request.
